# Development of Coconut Milk-Based Spicy Ice Cream as a Nondairy Alternative with Desired Physicochemical and Sensory Attributes

**DOI:** 10.1155/2021/6661193

**Published:** 2021-07-07

**Authors:** K. D. S. S. Perera, O. D. A. N. Perera

**Affiliations:** Department of Food Science and Technology, Faculty of Livestock, Fisheries and Nutrition, Wayamba University of Sri Lanka, Makandura, Gonawila, Sri Lanka

## Abstract

Spices have been a major influence on Sri Lankan cuisine since times immemorial. Spices are identified as one of the most distinctive ingredients for their indigenous flavor, aroma, and medicinal properties. In this study, coconut milk-based spicy ice cream was developed in compliance with the Sri Lankan standards to introduce a new perception of flavor using spices to the ice cream industry. Although coconut ice cream is commercially available in the local market, spicy flavored coconut ice cream is not yet available. Cinnamon (*Cinnamomum verum*), ginger (*Zingiber officinale*), and white pepper (*Piper nigrum*) are the spices used in the preparation of the ice cream as they are freely available and used as complementary spices in Sri Lanka. Physicochemical characteristics and sensory attributes of coconut milk-based spicy ice cream were compared with the existing normal coconut ice cream. In preparation of the ice cream, the same ice cream manufacturing process was followed with some modifications. Three different formulas (0.010%, 0.018%, and 0.025%) were developed by changing the percentage of spices added. The 0.018% spice-added sample was selected as the most acceptable ice cream with desired sensory attributes. pH (6.33 ± 0.01), titratable acidity (0.33 ± 0.05%), moisture (61.86 ± 0.33%), ash (0.41 ± 0.25%), total solids (38.02 ± 0.14%), overrun (66.76 ± 1.44%), protein (4.18 ± 0.16%), and fat content (11.66 ± 0.60%) were evaluated as physicochemical properties. Total phenolic content of the ice cream was expressed as 0.093 ± 0.002 mg gallic acid equivalents (GAE) per gram of sample in dry weight (mg/g). DPPH radical scavenging activity was 60.39 ± 0.02 mg ascorbic acid equivalents per gram of sample in dry weight (mg/g), and total antioxidant capacity was expressed as 0.36 ± 0.04 mmol ascorbic acid equivalent (AAE)/g of dry weight. Physicochemical properties of spicy coconut ice cream were more or less similar to that of normal coconut ice cream and in compliance with the Sri Lankan standards. Coconut milk-based spicy ice cream could be introduced to the market as a potential marketable nondairy product with spicy flavor, aroma, and smooth texture.

## 1. Introduction

Ice cream is a delicious, wholesome, and nutritious frozen dairy dessert, which is widely consumed globally, and it is very popular among people of all ages because of its taste and cool sensation property [1]. The main ingredients of ice cream are milk, cream, sweeteners, natural flavorings, and other optional ingredients such as eggs, nuts, fruits, chocolate, and candy. There are various types of ice creams available in the market including a wide range of flavors, colors, textures, and ingredients. Both the flavorings and the sweeteners are imparting sweet sensation to the ice cream. Sri Lanka is an Asian country which uses different flavorings in preparing meals, specially spices. Thus, Sri Lankans are more familiar with the flavor profiles of various spices. Spices also can be used as a flavoring for sweet products like ice creams [2]. Therefore, the main objective of this study was to formulate an ice cream with a new sensation which imparts both sweet and spicy tastes. Spices are the aromatic parts of tropical plants, dried seed, fruit, root, or a bark, traditionally used for flavoring, coloring, or preserving meals. Spices are consequential not only as foods but also as medicines. Cinnamon, ginger, and white pepper are complementary spices very well known among people due to their pleasant flavor, aroma, and medicinal value. Therefore, these three spices were selected to make the spicy ice cream. Cinnamon (*Cinnamomum verum*) is a spice obtained from the inner bark of several trees from the genus *Cinnamomum* that is used in both sweet and savory foods. Moreover, cinnamon is a powerful spice that has been used medicinally around the world for thousands of years [3]. Cinnamon bark contains several special compounds which are responsible for its many health-promoting properties, including cinnamaldehyde, cinnamic acid, and cinnamate. It has been revealed that special phenolic compounds, flavonoids, and antioxidants which are isolated from cinnamon are rich in antioxidant, antidiabetic, antimicrobial, immunity-boosting, and potential cancer and heart disease-protecting abilities [4]. Ginger (*Zingiber officinale*) is an underground stem or rhizome used as a flavoring and medicine in Asian and Arabic herbal traditions. Adding ginger as a flavoring makes dishes more delicious. Ginger is used as an ingredient for preparation of tea and production of some sweets and beverages [5]. It imparts many health benefits due to its antioxidants, antimicrobial activity, anti-inflammatory properties, and content of therapeutic compounds like gingerol, shogaol, paradol, and zingerone [6]. White pepper (*Piper nigrum*) is also one of the most popular culinary spices in the world. White pepper spice has had the black outer shell of the peppercorn removed, giving it a smooth mellow flavor. White pepper itself also contains immunity-boosting properties and anticancer, energy-boosting, anti-inflammatory, and antioxidant properties due to the compounds of capsaicin and piperine [7]. Though conventional ice cream is made by using dairy milk, over the years, nondairy milk has been more common, such as soy, almond, and coconut milk. Coconut milk ice cream may be an excellent alternative to those who suffer from lactose intolerance and benefit others as well. Coconut milk is a milky fluid obtained by manual or mechanical extraction of fresh coconut (*Cocos nucifera L*) kernel with or without addition of water. As a coconut-producing country, coconut milk plays a vital role in the Sri Lankan diet [8]. It is valued mainly for its characteristic nutty flavor and for its nutritional content. Coconut milk contains 56.3% moisture, 33.4% fat, 4.1% protein, 1.2% minerals, and 5.0% carbohydrates [9]. Development and quality evaluation of coconut milk-based soft ice cream has been reported [10]. Moreover, a study has been reported on physicochemical and sensory properties of ice cream formulated with virgin coconut oil [11]. However, the formulation of a spicy coconut ice cream and evaluation of its physicochemical and sensory attributes are yet to be investigated.

## 2. Materials and Methods

### 2.1. Preparation of Spices

#### 2.1.1. Preparation of Cinnamon Powder

Cinnamon quills (7 g) were purchased from a supermarket and washed using hot water (45°C) and dried at room temperature. Then, dried cinnamon quills were finely ground to obtain a powder with the help of a grinder (Philips HL1606, 500 W).

#### 2.1.2. Preparation of White Pepper Powder

Fresh white peppercorns (4 g) were purchased from a supermarket and cleaned using hot water (45°C) and dried at room temperature. Dried peppercorns were finely ground using a grinder (Philips HL1606, 500 W) to obtain the white pepper powder.

#### 2.1.3. Preparation of Ginger Extract (Water Extraction)

Fresh ginger rhizomes (15 g) were purchased from a supermarket. A mixture of 200 g of fresh, peeled, and cleaned ginger roots and 100 mL of filtered hot water (45°C) was blended using a medium speed level for 2 minutes using a blender (Wipro ANGEL WAM-L-55). Then, the blended mixture was filtered using a muslin cloth.

### 2.2. Ice Cream Production

Mature fresh coconuts were split and scraped with the use of a stainless steel electric coconut scraper (Walvia, India). Milk was extracted by blending the scraped coconut with water (coconut : water = 3 : 1). 360 g sugar, 10 g ice cream stabilizer Cremodan (Cuisine tech), and 14 g gelatin (Motha Confectionery) as a thickening agent were mixed together and added to the coconut milk (1.4 mL) [9]. The mixture was heated to 40°C, and spices were added separately.

Heating was continued to 90°C, and salt (8 g) was added. The mixture was pasteurized at 90°C for 20 minutes and cooled to room temperature. The mixture was filtered and homogenized. Then, it was kept for around 4 hours for aging. The mixture was fed into the instant ice cream-making machine (Softy line; capacity: 20 L/h). Ice cream was filled into a clean container (3 L) and stored in a freezer at -18°C prior to analysis.

### 2.3. Physicochemical Analysis of Spicy Coconut Ice Cream

#### 2.3.1. pH of Ice Cream

The pH of the melted ice cream sample was measured by using a pH meter (Starter 3000) [12].

#### 2.3.2. Titratable Acidity of Ice Cream

Titratable acidity of the melted ice cream sample was calculated as the percentage of lauric acid was determined by titration with 0.1 N sodium hydroxide [13].

#### 2.3.3. Moisture Content of Ice Cream

Moisture content of the spicy coconut ice cream and normal coconut ice cream (control) was measured according to the oven-dried method 925.10 as described in AOAC [14].

The empty moisture cans and lids were dried in the oven (Mammoth UF55, +20°C to +300°C) at 105°C for 3 hours and transferred to the desiccator to cool. The weight of cans with lids was taken. About 5 g each of spicy ice cream and control sample was weighed to the dishes, and samples were spread uniformly. The cans with samples were placed in the oven overnight.

After drying, the dishes with partially covered lids were transferred to the desiccator to cool.

Finally, the cans and samples were reweighed.

Calculation [14]:

(1) $$\begin{array}{c} \text{Moisture}\%=\frac{\left(W_{2}-W_{1}\right)\times 100\;}{\;W_{1}}, \end{array}$$

where *W*_1_ is the weight (g) of the sample before drying and *W*_2_ is the weight (g) of the sample after drying.

#### 2.3.4. Ash Content of Ice Cream

Ash content of spicy coconut ice cream and control samples was determined using the dry ash method 925.10 as described in AOAC [14].

The crucibles and lids were placed in the muffle furnace (Hobersal Rex C 700) at 550°C for 5 hours to ensure that impurities on the surface of the crucible were burned off. The crucibles were cooled in a desiccator. Then, the clean and dry crucibles and lids were weighed.

5 g each of dried spicy ice cream and control samples was weighed into crucibles and placed in the furnace without covering with the lids. The sample was incinerated at 550°C for 5 hours. The lids were placed after completed heating to prevent loss of fluffy ash. After cooling down in the desiccator, ash with crucibles and lids was weighed.

Calculation [14]:

(2) $$\begin{array}{c} \text{Ash }\left(\%\right)\text{ in dry basis}=\frac{W_{1}\times 100}{W_{2}}, \end{array}$$

where *W*_1_ is the weight (g) of ash and *W*_2_ is the weight (g) of the dried sample.

#### 2.3.5. Total Solids of Ice Cream (Gravimetric Method)

Both spicy ice cream and control samples were subjected to the following testing. Samples were transferred to a beaker and gradually warmed in a water bath (35-40°C). Then, samples were cooled to room temperature. Dishes and lids were heated in an oven at 102°C for 1 hour. The lids were placed on the dishes and immediately transferred to the desiccator to cool. Then, weights were taken. About 5 mL of spicy ice cream and control samples was poured into the dishes separately. Lids were placed, and weights were taken. The dish was placed in boiling water bath without a lid (bottom of the dish, directly heated by steam). Heating was continued till most of the water is removed. The dish was removed from the water bath and placed in the oven at 102°C for 2 hours alongside lids. Thereafter, lids were placed and kept in a desiccator to cool.

The weights were taken, and dishes were heated again with lids alongside in an oven for 1 hour. Then, lids were placed and transferred immediately to the desiccator, and weights were recorded [14].

Calculation [14]:

(3) $$\begin{array}{c} \text{Total solid content}=\frac{M_{2}-\text{Mo}\times 100}{M_{1}-\text{Mo}}, \end{array}$$

where Mo is the mass in g of the dish, *M*_1_ is the mass in g of dish + lid + sample, and *M*_2_ is the mass in g of dish + lid + dried sample.

#### 2.3.6. Melt Down of Ice Cream

Melt down of spicy coconut ice cream and control samples was determined according to method 941.08 as described in AOAC [14].

Melt down of ice cream samples was evaluated using a 50 g ice cream block (height: 2 cm, diameter: 6 cm) which was placed on a metric test sieve that was supported by a previously weighed beaker. The mass of melted ice cream collected in the beaker was recorded at 5-minute time intervals for a 60-minute duration. The mass of melted ice cream (g) was plotted against the time (min) [14].

#### 2.3.7. Overrun of Ice Cream

Overrun of the ice cream was determined by using about 20 mL of ice cream mix and frozen ice cream.

Calculation [14]:

(4) $$\begin{array}{c} \frac{\text{Overrun}=\text{volume of the ice cream}–\text{volume of the ice cream mix}\times 100}{\text{Volume of the ice cream mix}}. \end{array}$$

#### 2.3.8. Crude Protein Content of Ice Cream

Crude protein content of the spicy coconut ice cream and control was determined by the Kjeldahl method: 920.87 as described in AOAC [14].

Approximately 0.5-1.0 g of dried ice cream sample was placed in a dry and cleaned digestion flask. 25 mL of concentrated sulfuric acid and Kjeldahl tablet were added to the digestion flask. Digestion was run for 3 hours using a mini Kjeldahl unit (Block Digestion Unit Model: K-424). After, the digestion flask was allowed to cool and conducted distillation using an automated distillation unit (BUCHI, USA). Finally, the sample was titrated with 0.25 N HCl solution, and crude protein content was determined (*N*∗6.25). 0.25 N HCl was standardized by titrating 0.25 N sodium carbonate solution.

#### 2.3.9. Crude Fat Content of Ice Cream

Fat content of the spicy coconut ice cream and control was determined by the Soxtherm method as described in AOAC [14].

Oven-dried samples were used to measure crude fat using a fat analyzer (Model: SER 148, Italy). 1 g of sample (*W*_1_) was weighed accurately by using analytical laboratory balance in a weighed extraction thimble (*W*_2_). The rubber ring was selected, and thimbles were placed in a fat analyzer. 80 mL of petroleum ether was filled into each tube. Then, the fat analyzer was programmed for 5 minutes in immersion, 20 minutes in washing, and 30 minutes in recovery.

After completion of the process, the crucible was oven-dried at 105°C for 6 hours and reweighed [14]:

(5) $$\begin{array}{c} \text{Fat }\left(\%\right)=\frac{W_{3}-W_{2}\times 100}{W_{3}\times \text{DM}}. \end{array}$$

### 2.4. Antioxidant Activity of Ice Cream

Ice cream samples were dried by lyophilization (Martin Christ, Freeze Dryer, Alpha 1-2/LD Plus) for 24 h. An ice cream sample (13 g) was mixed with a mixture of 70 mL methanol/water (80/20, *v*/*v*) and vortexed at high speed for 30 minutes. Then, the mixture was centrifuged (Hettich, EBA 20) for 10 min at 792 rpm. After the centrifugation, the extracts were subsequently filtered through a filter paper (Whatman No. 42; Whatman Paper Ltd, Maidstone, UK). Finally, the prepared extracts were evaporated in a rotary evaporator (Hahnvapor, Model HS-2005 V, Hahnshin Scientific, Korea) at 40°C under vacuum and stored at -18°C until assayed within 1 week [15].

#### 2.4.1. Total Phenolic Content

About 1 mL of the melted ice cream sample was added to a 1.5 mL diluted Folin-Ciocalteu reagent. Samples with the reagent were left for 5 minutes, and then, 1 mL 7.5% sodium carbonate (*w*/*v*) was added. The samples were vortexed and kept in the dark for 2 hours. The absorbance was measured at 760 nm using a spectrophotometer (Optima, SP-3000, Tokyo, Japan). The calibration curve of gallic acid was plotted to evaluate the activity capacity of the samples. The result was expressed as milligram of gallic acid equivalents (GAE) per gram of ice cream sample (mg GAE/100 g of dry weight) [16].

#### 2.4.2. DPPH Radical Scavenging Activity

About 3 mL of prepared DPPH solution was added to 1 mL of melted ice cream sample. Then, solutions in the test tubes were shaken well. Samples were incubated in the dark for 15 minutes at room temperature. Finally, the absorbance was measured at 517 nm using a spectrophotometer (Optima, SP-3000, Tokyo, Japan) [16].

The DPPH radical scavenging activity (%) was calculated as

(6) $$\begin{array}{c} \text{DPPH radical scavenging activity }\left(\%\right)=\frac{\;\left[A_{\text{c}}-A_{\text{s}}\right]\times 100}{A_{\text{c}}}, \end{array}$$

where *A*_c_ is the absorbance of the control and *A*_s_ is the absorbance of the sample [16].

#### 2.4.3. Total Antioxidant Capacity

About 0.1 mL of the ice cream sample was added into a test tube and mixed with 1 mL of reagent solution (0.6 M sulfuric acid, 28 mM sodium phosphate, and 4 mM ammonium molybdate).

The tube was capped with an aluminum foil and incubated at 95°C for 90 minutes. Then, the tube was cooled down to room temperature, and the absorbance was measured at 695 nm using a spectrophotometer (Optima, SP-3000, Tokyo, Japan) [16].

### 2.5. Sensory Evaluation

The acceptability of the coconut milk-based spicy ice cream was evaluated by conducting a sensory evaluation with 30 untrained, both male and female panelists, in the age between 22 and 30 years. The sensory attributes tested were color and appearance, body and texture, aroma and flavor, melting quality, and overall acceptability. A 9-point hedonic scale was used. The values were scored on least preference (1) to most preference (9).

### 2.6. Statistical Analysis

MINITAB version 14 was used to statistically analyze all the results obtained from physicochemical and sensory evaluation. A one-way analysis of variance (ANOVA) was performed, and the significant difference was defined at *p* < 0.05 for the results of all analyses with both control and coconut spicy ice cream. The final results obtained were expressed as the mean values ± standard deviation of three replicates. Sensory evaluation data were analyzed using the Kruskal-Wallis test.

## 3. Results

### 3.1. Selection of the Most Acceptable Spicy Ice Cream Formula

**Figure 1:**
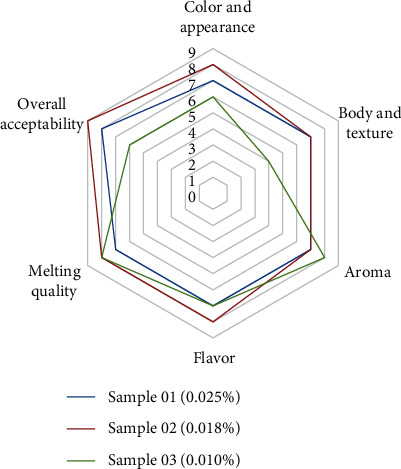
Graphical design for sensory analysis of coconut milk-based spicy ice cream.

Data obtained from sensory evaluation revealed that sample 02, containing 0.018% *w*/*w* of spices, is the most acceptable formula. Color and appearance, body and texture, spicy flavor and aroma, and overall acceptability of sample 02 were higher than that of 0.025% spice-incorporated sample 01 and 0.010% spice-incorporated sample 03. Melting quality of sample 02 is lower than that of samples 01 and 03 (Figure 1).

### 3.2. Physical Properties of Ice Cream

There is a significant difference in pH between spicy coconut ice cream and normal coconut ice cream (Table 1).

pH of the spicy coconut ice cream is lower than the pH of the normal coconut ice cream. There is no any significant difference (*p* > 0.05) between moisture content, titratable acidity, and overrun of the spicy coconut ice cream when compared to the normal coconut ice cream.

**Figure 2:**
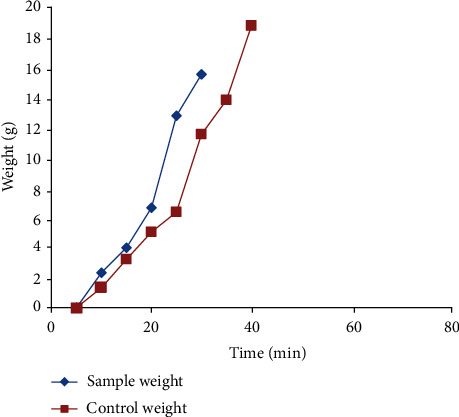
Comparison of melting rates of the spicy coconut ice cream with normal coconut ice cream.

The melting rate of spicy coconut ice cream is higher than that of normal coconut ice cream (Figure 2).

### 3.3. Chemical Properties of Ice Cream

There is no any significant difference (*p* > 0.05) between fat content, protein content, total solids, and ash content of the spicy coconut ice cream when compared to the normal coconut ice cream (Table 2).

### 3.4. Antioxidant Properties

There is a significant difference (*p* < 0.05) in total phenolic content and DPPH radical scavenging activity of spicy coconut ice cream when compared with normal coconut ice cream. There was no any significant difference (*p* > 0.05) in the total antioxidant capacity of spicy ice cream when compared to the normal coconut ice cream. Total phenolic content and DPPH radical scavenging activity of spicy coconut ice cream are higher than that of normal coconut ice cream (Table 3).

### 3.5. Shelf Life Studies

Total plate count (TPC) in both spicy coconut ice cream and normal coconut ice cream was increased with the storage time. There were no any yeast and mold counts (Table 4).

## 4. Discussion

This study revealed that the spicy coconut ice cream which consolidated 0.018% spices (sample 02) demonstrated a higher mean rank for the five attributes, in particular, color and appearance, body and texture, spicy flavor, aroma, and overall acceptability from sensory evaluation (Figure 1). Compared to the normal coconut ice cream, these study findings indicate that there were some similitudes and furthermore a few contrasts in the outcomes for physicochemical properties and chemical properties of spicy coconut ice cream; however, the results comply with the recommended limits for ice cream of Sri Lankan standards.

Moisture content of both ice creams was similar. Moisture basically comes to the ice cream by milk and maintained through the freezing process. Since the same quantity of coconut milk was used to make both ice creams and the same freezing conditions (continuous freezing) were used, moisture content of both ice creams was similar. The recommended moisture content for ice cream is 61.7% [17], and spicy coconut ice cream contained moisture around the recommended value of 61.86 ± 0.33%.

Ash content of spicy coconut ice cream (0.41 ± 0.25%) and normal coconut ice cream (0.38 ± 0.05%) was not considerably different because the addition of spices in minor concentrations did not alter the ash content of spicy ice cream. Moreover, the result lies within the recommended range for ash content (0.3-0.6%).

Titratable acidity of dairy ice cream expressed as lactic acid is ranged from 0.19 to 0.22% [18] that complied with Sri Lankan standards. Lauric acid, present in coconut milk, is reasonable for the natural acidity of coconut ice cream. According to the results obtained from this study, titratable acidity of spicy coconut ice cream (0.33 ± 0.05%) and normal coconut ice cream (0.42 ± 0.08%) was not significantly different. The acidity value of coconut ice cream was higher than the standard value because cow milk contains 4.8 to 5.1% lactose and coconut milk contains 48% lauric acid. As observed, the pH of spicy coconut ice cream (6.23 ± 0.01) was lower than the pH of normal coconut ice cream (6.38 ± 0.02) because the addition of spices increases milk solids nonfat (MSNF) which raises the acidity and lowers the pH. Basically, the pH value of normal ice cream is about 6.2-6.3 [19]. Therefore, the pH value of spicy coconut ice cream was within the recommended range.

According to the results, the percentage of total solids of spicy coconut ice cream was similar to the total solid percentage of normal coconut ice cream because the same fat source was used in the ice cream mix. Ice cream with fat content of 12-15% usually consisted of 32-40% of total solid content [20]. Based on the formulations of ice cream mix, spicy coconut ice cream contained 38.02 ± 0.14% of the total solid content, and this value falls within the recommended range.

The overrun of spicy coconut ice cream was indicated as 66.76 ± 1.44%, and it falls within the recommended range (60-90%) [20]. The overrun of ice cream is affected by fat source and fat content [21]. As observed, there was no any significant difference in overrun of spicy coconut ice cream compared to the normal coconut ice cream as coconut milk was the major fat source used for both.

From the melt down curves (Figure 2), it can be observed that the melting rate of spicy coconut ice cream was higher than that of normal coconut ice cream. A low freezing point is the primary cause of rapid melting whilst a high amount of air and fat incorporation (overrun) tends to slow down the melting [22]. The existence of more or less similar overrun in both types of ice creams implies that the overrun does not affect the melting rate. Due to the incorporation of spices, the structure of ice cream mix was unstable in spicy ice cream than the normal coconut ice cream; therefore, spicy coconut ice cream showed a higher melting rate.

Spicy coconut ice cream and normal coconut ice cream contain fat content of 11.66 ± 0.66% and 11.06 ± 0.38%, respectively. This property indicates that both ice creams have similar fat contents as coconut milk plays a role as the major fat source. Recommended fat content of an ice cream is 8-12% [20], and spicy ice cream lies within the range.

According to the results, protein content of both ice creams was not significantly different. Results indicate that the protein content of spicy coconut ice cream (4.18 ± 0.16%) was higher than the recommended protein content of ice cream (4%) [23]. The Kjeldahl method involved the total nitrogen determination and followed by the conversion of total nitrogen into crude protein content using a suitable conversion factor, 6.38. The presence of nitrogen might come from nonprotein components as well [24]. Therefore, this may be the probable reason for the detected increase in protein content of ice cream as spices were used.

An antioxidant is a major bioactive compound present in spices. In this study, total phenolic content, DPPH radical scavenging activity, and total antioxidant capacity were analyzed to examine the antioxidant activity of ice cream. The developed spicy coconut ice cream contained higher values for all three antioxidant properties compared to normal coconut ice cream as 0.093 ± 0.002 (mg gallic acid equivalent/g of dry weight), 60.39 ± 0.02 (mg ascorbic acid equivalent/g of dry weight), and 0.36 ± 0.04 (mmol ascorbic acid equivalent/g of dry weight), respectively, due to addition of spices. Cinnamon contains cinnamaldehyde and cinnamic acid, ginger contains gingerol, and white pepper contains piperine as the phenolic compounds. Polyphenols have the ability to quench DPPH radicals by providing hydrogen atoms or by electron donation. The higher the percentage of inhibition of free radical activity, the more potent the antioxidant activity of the extract in terms of hydrogen atom donating capacity.

Total plate count reflects the viable microbial population. It reveals the microbiological quality of the product examined and also reflected on the quality of raw materials, degree of hygiene, and the cleanliness maintained during manufacture, handling, and storage of the product. In the four weeks of study, all the total plate counts were less than 500 per 1 mL, which is given in the SLS 223 [25]. As shown in Table 4, there were no any yeast and mold counts as well. Yeasts are capable of growing well at low pH and can produce off flavors. Molds can grow well on the surfaces of milk-based products when oxygen is present, with the low pH being selective for them. But frozen conditions in ice cream prevent the growth of yeast and mold in ice cream. Therefore, in four weeks of time, the product was safe with no microorganisms.

## 5. Conclusion

An ice cream was developed using coconut milk as an alternative for cow milk with the incorporation of locally available spices (cinnamon, ginger, and white pepper) as a novel product. Among the developed three different formulas, 0.018% spice-incorporated sample 02 achieved higher overall sensory acceptability. This spicy coconut ice cream contained considerably similar values for physicochemical properties compared to normal coconut ice cream but potentially higher antioxidant properties when compared with the normal coconut ice cream. Accordingly, coconut milk-based spicy ice cream can be introduced to the market as a nondairy product with desired sensory attributes and functional properties.

## Figures and Tables

**Table 1:** Comparison of physical properties of spicy coconut ice cream with control.

Property	Spicy coconut ice cream	Normal coconut ice cream (control)	*p* value
Moisture content (%)	61.86 ± 0.33^a^	62.73 ± 0.54^a^	0.077
pH	6.23 ± 0.01^a^	6.38 ± 0.02^b^	0.000
Titratable acidity (%)	0.33 ± 0.05^a^	0.42 ± 0.08^a^	0.176
Overrun (%)	66.76 ± 1.44^a^	71.59 ± 4.56^a^	0.155

^∗^Values are the mean ± SD of three replicates. ^∗^Means with a different letter within a column are significantly different (*p* < 0.05).

**Table 2:** Comparison of chemical properties of spicy coconut ice cream with normal coconut ice cream.

Property (%)	Spicy coconut ice cream	Normal coconut ice cream	*p* value
Fat content	11.66 ± 0.60^a^	11.06 ± 0.38^a^	0.215
Protein content	4.18 ± 0.16^a^	4.07 ± 0.26^a^	0.584
Total solids	38.02 ± 0.14^a^	37.75 ± 0.44^a^	0.070
Ash content	0.41 ± 0.25^a^	0.38 ± 0.05^a^	0.120

^∗^Values are the mean ± SD of three replicates. ^∗^Means with a different letter within a column are significantly different (*p* < 0.05).

**Table 3:** Comparison of antioxidant activities of spicy coconut ice cream with normal coconut ice cream.

Antioxidant property	Spicy coconut ice cream	Normal coconut ice cream	*p* value
Total phenolic content (mg gallic acid equivalents (GAE)/g of dry weight)	0.093 ± 0.002^a^	0.072 ± 0.003^a^	0.001
DPPH radical scavenging activity (mg ascorbic acid equivalent/g of dry weight)	60.39 ± 0.02^a^	57.4 ± 0.1^a^	0.000
Total antioxidant capacity (mmol ascorbic acid equivalent (AAE)/g of dry weight)	0.36 ± 0.04^a^	0.32 ± 0.03^b^	0.238

^∗^Values are the mean ± SD of three replicates. ^∗^Means with a different letter within a column are significantly different (*p* < 0.05).

**Table 4:** Changes in microbial counts of spicy coconut ice cream and normal coconut ice cream during storage.

Sample	Test		Storage time (weeks)		
		01	02	03	04
Spicy coconut ice cream	TPC	260	316	310	410
	YMC	0	0	0	0
Ordinary coconut ice cream	TPC	377	410	414	452
	YMC	0	0	0	0

## Data Availability

All data generated or analyzed during this study are included in this published article.
